# Frequency and spectrum of M_2_ mutants and genetic variability in cyto-agronomic characteristics of fenugreek induced by caffeine and sodium azide

**DOI:** 10.3389/fpls.2022.1030772

**Published:** 2023-01-16

**Authors:** Neha Naaz, Sana Choudhary, Nidhi Sharma, Nazarul Hasan, Najla A. Al Shaye, Diaa Abd El-Moneim

**Affiliations:** ^1^ Department of Botany, Aligarh Muslim University, Aligarh, UP, India; ^2^ Department of Biology, College of Science, Princess Nourah bint Abdulrahman University, Riyadh, Saudi Arabia; ^3^ Department of Plant Production (Genetic Branch), Faculty of Environmental Agricultural Sciences, Arish University, El-Arish, Egypt

**Keywords:** fenugreek, chemical mutagens, cytological abnormalities, mutagenic effectiveness and efficiency, M2 mutants, SEM

## Abstract

*Trigonella foenum graecum* L. (Fenugreek) is a valuable medicinal plant cultivated for decades for its therapeutic characteristics. Still no pronounced improvement concerning wild form was accomplished as it is a self-pollinating crop. Induced mutagenesis is encouraged as a remarkable tool on this plant to circumvent the genetic bottleneck of cultivated germplasms. As a result, novel allelomorphic combinations for short-term agronomic attributes were developed. Fenugreek cultivar Pusa Early Bunching, selected for the present experiment, was mutagenized with five doses (0.2%, 0.4%, 0.6%, 0.8%, and 1.0%) of caffeine and sodium azide (SA) to evaluate its impact on the qualitative and quantitative traits of M_1_ and M_2_ generation conducted in a Complete Randomized Block Design (CRBD), replicated five times during 2019–2020 and 2020–2021, respectively. The frequency of induced phenotypic variations was assessed in M_2_ progenies, resulting in the identification and isolation of a broad spectrum of mutants with altered phenotypes. Mutagenic effectiveness and efficiency were found to be maximum at lower concentrations of the mutagen treatments and highest in SA, followed by caffeine. Various morphological mutants with modified characters were observed at different concentrations in M_2_ generation. The spectrum of mutations was wider in SA than in caffeine, as caffeine produced 51 while SA produced 54 individual mutants under seven major categories. The maximum frequency of morphological mutants was associated with leaf, followed by plant size, plant growth habit, pod, seed size, seed shape, and seed color. Morphological and structural variations in the guard cells of stomata and seeds were observed through scanning electron microscopy. The variations created in the economically important traits may enrich the genetic diversity of this plant species. Moreover, these morphological mutants may serve as a source of elite genes in further breeding programs of fenugreek.

## Introduction

**Figure 1:**
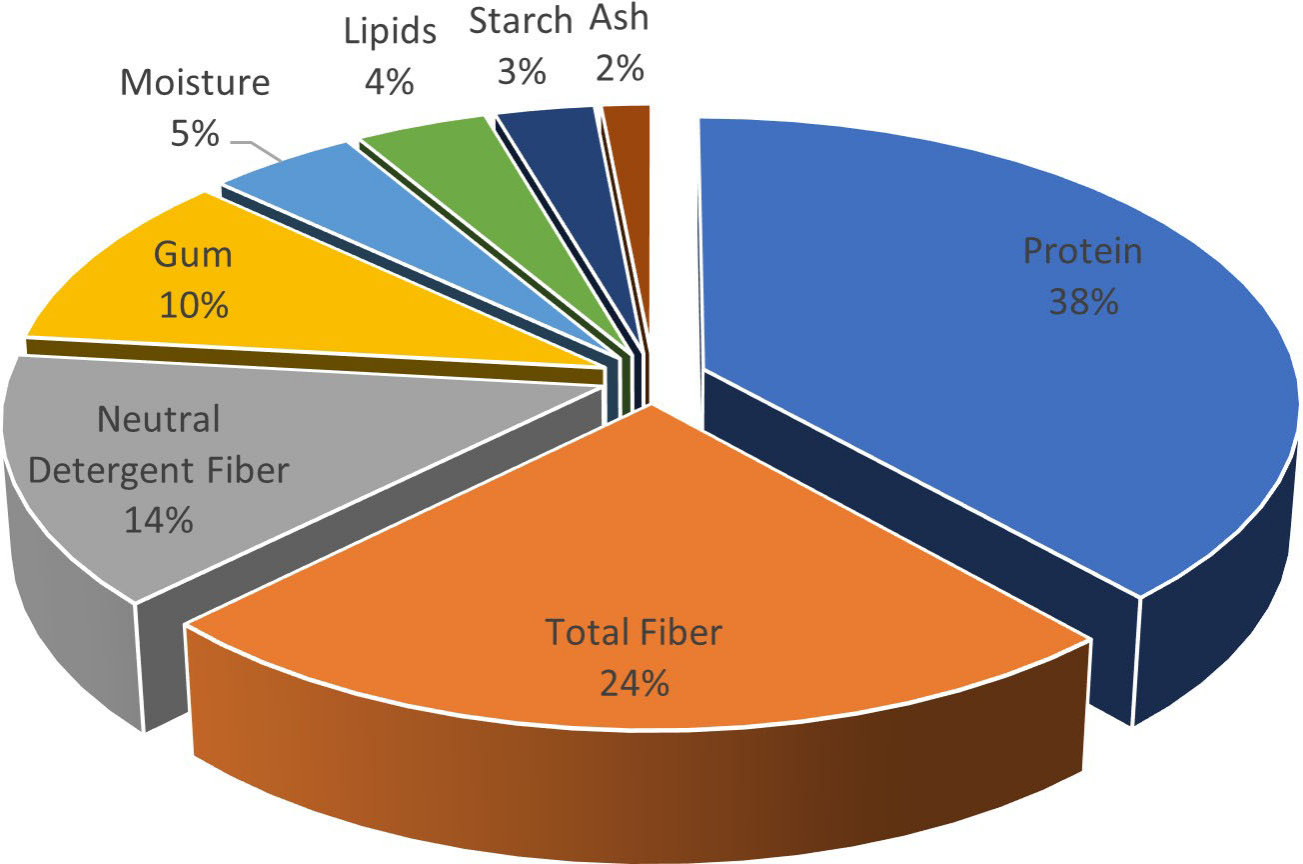
Proximate composition of fenugreek seeds.

Burgeoning demand and consumption of herbal remedies result in shortages and even the exhaustion of some medicinal plants, which destroys genetic diversity and habitats (Canter et al., 2005; Wang et al., 2020). The current global herbal trade is evaluated to be the US$ 62 billion and is estimated to grow to US$ 5 trillion by 2050 (Kumar and Gupta, 2008). Medicinal and aromatic plants (MAPs) are valuable resources of plant secondary metabolites, including phenolics, flavonoids, and alkaloids for human healthcare (Nile et al., 2017). Mutation breeding creates a practical framework for enhancing the yield and quality of secondary metabolites and supplementing existing germplasm for crop improvement (Basu et al., 2008; Kaiser et al., 2020). Fenugreek is a self-pollinating, dicotyledonous, aromatic herb of the family Fabaceae with a diploid number of chromosomes (2*n* = 16) commonly known as “Methi”. Fenugreek cultivar Pusa Early Bunching (PEB) was the best variety for more herbage parameters resistant to downy mildew and rots. It matures in 125 days from seed to seed. Fenugreek is among the oldest medicinal plants in human history, as its leaves and seeds possess therapeutic properties (Hilles and Mahmood, 2021). Fenugreek is a multipurpose cash crop consumed as a leafy vegetable, with dried leaves as an herb and seed as a spice. Fenugreek contains 33 mg of iron per 100 g dry weight, making it a good source of iron. Calcium, iron, phosphorus, riboflavin, carotene, thiamine, niacin, and vitamin C are all vitamins and minerals found in the leaves (Visuvanathan et al., 2022). Fenugreek holds several bioactive phytochemicals such as trigonelline (alkaloid), diosgenin (steroidal sapogenin), and mucilage, which are identified for their protective roles in the plant (Zandi et al., 2014; Ashrafi Parchin et al., 2021). Its grains are an essential source of carbohydrates (mainly galactomannans), proteins (lysine and tryptophan), fixed oils (lipids), alkaloids (trigonelline), flavonoids (ornithine, viticsine, and quercetin), free amino acids (4-hydroxyisoleucine), essential nutrients (calcium, iron, and beta-carotene), steroidal saponins (diosgenin, ticogenine, and neoticogenine), choline, vitamins A, B, C, and nicotinic acid (Hanafy and Akladious, 2018; Kavina et al., 2020). The (**Figure 1**) shows the main composition of the fenugreek seeds (Pal and Mukherjee, 2020). The oil of fenugreek is rich in linoleic acid (42.71%–42.80%), linolenic acid (26.03%–26.15%), and oleic acid (14.24%–14.40%) (Sowmya and Rajyalakshmi, 1999). An alkaloid, trigonelline, isolated from fenugreek seeds possesses several pharmacological effects such as anti-diabetic, anticarcinogenic, hypoglycemic, serum lipid-lowering, antiseptic, and hypocholesterolemic, though diosgenin belonging to the triterpenes is used as natural birth control remedy (Beyzi et al., 2021). Pharmacologically, this plant possesses anti-diabetic, anti-inflammatory, diuretic, anti-bacterial, anti-bloating, antioxidant, anti-diarrhea, anti-anorexia, anti-rheumatoid, anticarcinogenic, and neuroprotective effects (Ashrafi Parchin et al., 2021; Hilles and Mahmood, 2021).

**Figure 2:**
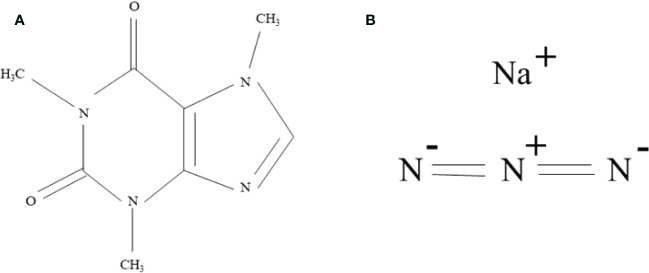
Chemical structure of **(A)** caffeine (C_8_H_10_N_4_O_2_) and **(B)** sodium azide (NaN_3_).

Genetic variability is an essential tool in crop improvement programs, as it provides a variety of variants for improved selection through mutation, hybridization, recombination, and selection techniques (Dhumal and Bolbhat, 2012). MAPs have a narrow genetic variation. In this regard, induced mutagenesis is a practical approach for expanding genetic variability *via* mutagen to raise desirable mutants with improved yield and agronomic traits (Roychowdhury et al., 2012; Asif et al., 2019). In mutation breeding, the choice of an efficacious mutagen is crucial to create beneficial mutants (Roychowdhury and Tah, 2011). Over the decades, mutagens have been beneficial in inducing phenotypic variations (Kavina et al., 2020). Caffeine (1,3,7-trimethylxanthine), a purine alkaloid, arises naturally in coffee (*Coffea*), tea (*Camellia senensis*), *etc*. (Alkhatib et al., 2018). As a result, interaction with DNA alters some physical properties (DNA denaturation) leading to the high frequency of spontaneous mutations, hindering DNA repair mechanism, and triggering DNA–DNA or DNA–protein linking (Aslam et al., 2017). Fries and Kihlman (1948) observed the mutagenic effect of caffeine on *Ophiostoma multiannulatum.* Moreover, it can act synergistically to produce chromosomal abnormalities in mammalian cells (Khursheed et al., 2009). Sodium azide (NaN_3_) is the most effective mutagen in crop plants (**Figure 2**). The mutagenicity of sodium azide is facilitated *via* the development of azide compound which enters the nucleus, interacts with DNA, and generates point mutation in the genome (Al-Qurainy, 2009; Ingle et al., 2018; Chaudhary et al., 2021). This mutagenicity was noticed in many crops such as lentil (Khan et al., 2006), fenugreek (Siddiqui et al., 2007), chickpea (Kulthe and Kothekar, 2011), wheat (Srivastava et al., 2011), tomato (Abdulrazaq and Ammar, 2015), *etc*. Several workers reported that a mutant population developed using SA in fenugreek (Bashir et al., 2012). The present research was conducted to hypothesize the mutagenic outcome of caffeine and sodium azide on M_1_ and M_2_ generation of *Trigonella foenum graecum* L. cultivar PEB.

## Materials and methods

Certified, healthy, and dry fenugreek cultivar PEB germplasm was procured from the Indian Agricultural Research Institute, New Delhi. This experiment employed caffeine and sodium azide (NaN_3_) as a mutagen. The selected uniform-sized seeds (10% moisture) were presoaked in distilled water for 12 h to improve the efficacy of mutagen. Then, a stock solution (v/v) of chemical mutagens was prepared, and pH was maintained through buffer tablets (pH 7.0). The seeds were treated with caffeine and SA (0.2%, 0.4%, 0.6%, 0.8%, and 1.0%) doses for 9 h with constant intermittent shaking to provide adequate aeration to the seeds at room temperature. A total of 100 untreated seeds were maintained as control. Afterward, the treated seeds were rinsed carefully under running tap water to eliminate residual mutagen. The 100 seeds from each were immediately sown in five replicates conducted in a CRBD during mid-October of 2019–2020 to upraise the M_1_ population. The site of this experiment, Aligarh, has a semi-arid and subtropical climate with hot summers and cold winters. The average temperature range is 28–45°C in summer and 5–11°C in winter. The average annual rainfall is 800 mm. Most of the rainfall occurs from late June to early October, bringing high humidity. The soil is sandy loam and alkaline in nature. Later, standard cultural practices such as irrigation, fertilization, and weeding were carried out. The seeds of M_1_ plants were harvested concentration-wise and stored for raising the M_2_ generation.

### Mutant population development

In this study, all the seeds were harvested from each concentration of the M_1_ generation. Then, 100 healthy seeds were sown treatment-wise in plant progeny rows for raising the M_2_ generation during the rabi season, *i*.*e*., mid-October, 2020–2021. A total of 1,100 M_2_ seeds were sown, of which 854 germinated and 826 M_2_ plants survived. Mutants from the M_2_ population were isolated and screened based on visible phenotypic mutations. The mutations induced in the morphology of the cultivar were categorized into seven major categories, *viz*., plant size, plant habit, leaf, pod, seed size, seed color, and seed shape. The mutants isolated and screened had undergone further analysis.

### Parameters studied in M_1_ generation

#### Germination, plant survival, and pollen fertility

The seed germination was recorded right from the emergence of the first shoot in each treatment as well as control on every alternate day, until maximum germination was attained. The survival of plants was counted at the time of maturity. Fresh and young flowers from 20–30 randomly selected plants were taken from each treatment as well as the control. The pollens from mature and undehisced anthers were dusted on a slide containing a drop of 1% acetocarmine. Pollen grains which took stain and had a regular outline were considered as fertile, while those without stain and irregular in shape and size were considered as sterile. The percentage of germination, of plant survival, and of pollen fertility, respectively, were calculated by the following:


$$\text{Germination}\left(\%\right)=\frac{Number\;of\;seeds\;germinated}{Number\;of\;seeds\;sown}\times 100$$

$$\text{Survival}\left(\%\right)=\frac{Number\;of\;plant\;at\;maturity}{Number\;of\;seeds\;germinated}\times 100$$

$$\text{Pollen fertility}\left(\%\right)=\frac{Number\;of\;fertile\;pollen}{Total\;number\;of\;pollen}\times 100$$

#### Photosynthetic pigments

The photosynthetic pigments were calculated next to the pigment extracted from fresh leaves through 80% acetone (Mackinney, 1941). Fresh leaves (1 g) were macerated in 20 ml of acetone and then centrifuged at 5,000 rpm for 5 min. The supernatant procured is diluted with acetone and then made up to a final volume of 100 ml. The absorbance of chlorophyll content at 663 and 645 nm and carotenoids at 480 and 510 nm in respect of optical density, respectively, were recorded through a UV–vis spectrophotometer. It was calibrated as per the formula proposed by Arnon (1949).

#### Proline content

In total, 0.5 g of fresh leaves were homogenized in 5 ml of 3% sulfosalicylic acid for 5 mins to quantify proline content. The filtrate (2 ml) blended with reaction mixture (containing 2 ml of 1% (w/v) ninhydrin, 60% (v/v) glacial acetic acid as well as 20% (v/v) ethanol seethed for 1 h at 100°C. Then, the reaction concoction was stopped in an ice bath, with 4 ml of toluene added to each test tube. The organic phase containing toluene was extracted, and the absorbance of the red color was noted at 520 nm using pure proline as standard (Bates et al., 1973).


μmol/g tissue=μg proline/ml×toluene/115.5×5/g sample

#### Quantitative traits

The data for different agronomic traits were thoroughly evaluated, namely, shoot length (cm), root length (cm), pods bearing branches/plant, number of pods/plant, number of seeds/pod, 1,000 seed weight (g), and yield per plant (g) from the M_1_ population.

#### Meiotic analysis

For cytological analysis, young flower buds were collected separately from each treatment, controlled, fixed in freshly prepared Carnoy’s fluid (6:3:1 alcohol/chloroform/glacial acetic acid) for 24 h, then washed, and stored in 70% alcohol. The anthers were crushed in 2% acetocarmine for staining; permanent slide was prepared by dehydrating in butyl alcohol series mounted in Canada balsam, and it was dehydrated at 45°C.

Meiotic abnormalities (%) = *Total no. of meiotic abnormalities/Total no. of PMCs* * 1000

#### Total protein content, phenolic content, and flavonoids

Protein content was assessed in the protein samples by Lowry et al. (1951). Then, a spectrophotometer was used to measure the total protein content at 750 nm.

The phenolic content in the extracts was evaluated with the Folin–Ciocalteu colorimetric procedure based on gallic acid equivalent as demonstrated by Chandra et al. (2014). The absorbance was spectrophotometrically recorded at 750 nm and stated as milligrams of gallic acid/gram fresh weight (FW).

Fresh leaves (0.5 g) were blended with 10 ml of 80% methanol, and the extract was filtered to determine the total flavonoid content. Then, 4 ml distilled water and 0.3 ml of 5% NaNO_2_ were added to 1 ml of the extract. Subsequently, 0.3 ml of 10% AlCl_3_ was blended in, and the mixture was set aside to incubate for 6 min, succeeded by adding 2 ml of 1 M NaOH. The solution was diluted with water up to a final volume of 10 ml and vigorously shaken. The absorbance was spectrophotometrically estimated at 430 nm, utilizing quercetin as the standard and stated as milligram of quercetin/gram FW.

### Parameters studied in M_2_ generation

Mutation frequency:


$$\%\;mutated\;plant\;progeny\;(Mp)=\frac{No.\;of\;mutant\;plant\;progrenies\;segregating\;in\;M2}{Total\;no.\;of\;M1\;plant\;progenies}$$

The following formula for calculating mutagenic effectiveness and efficiency was suggested by Konzak et al. (1965).


$$\text{Mutagenic effectiveness rate for chemical mutagen }=\frac{Rate\;of\;mutation\;(Mp)}{Concentration\times duration\;of\;treatment}$$

$$\text{Mutagenic efficiency}=\frac{Rate\;of\;mutation\;(Mp)}{Biological\;damage\;in\;M1\;generation}$$

Biological damage was measured based on meiotic abnormalities.

#### Analysis of the stomatal aperture of leaves and micromorphology of seeds using scanning electron microscopy

Scanning electron microscope imaging was employed to analyze the stomatal aperture and was executed according to Hasan et al. (2022a). The same protocol was followed for imaging seeds to characterize the shape and surface topology examined using JEOL-SEM (Japan) at University Sophisticated Instruments Facility, Aligarh Muslim University, Aligarh. Then, micrographs were acquired from a scanning electron microscope at 15 kV.

#### Statistical analysis

The data obtained are presented as mean ± SE of five replicates and command to one-way ANOVA. The treatment means were compared using Duncan Multiple-Range Test at *p* <0.05 carried out by SPSS 16 software.

## Results

For the recognition of variants and mutants, the plants upraised from mutagenized seeds were correlated with control throughout their growth. The present investigation evaluated the comparative effects of caffeine and SA on the undertaken parameters of fenugreek during M_1_ and M_2_ generation, and these were analyzed statistically.

### M_1_ generation observations

**Figure 3:**
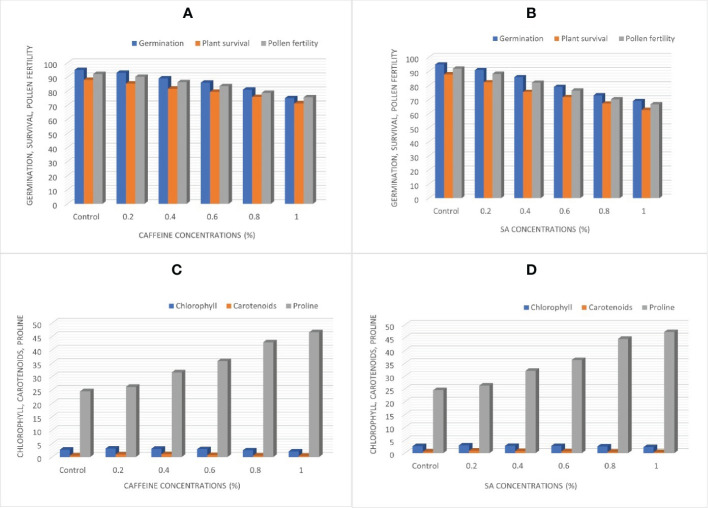
**(A, B)** Germination, plant survival, and pollen fertility. **(C, D)** Chlorophyll, carotenoids, and proline content, affected by caffeine and sodium azide during M_1_ generation.

There was a gradual decline in germination, survival, and pollen fertility percentage of fenugreek plants induced from caffeine, and this is displayed in **Figures 3A, B**. The obtained results negatively correlate with the mutagen doses compared with the control. The maximum seed germination percentage was 95.00% in the control, significantly decreasing to 75.00% and 69.00% in the 1.0% treatment of caffeine and SA, respectively. Therefore, germination was most affected at higher doses (**Figures 3A, B**). There was a progressive decline in the percentage of survival of plants at maturity with 88% (control) decreasing from 85.35% (0.2%) to 71.26% (1.0%) in caffeine treatments (**Figure 3A**). In SA treatments, it decreases from 82.30% (0.2%) to 62.70% (1.0%) (**Figure 3B**). The highest dose of 1.0% of both caffeine and SA treatments resulted in a minimum percentage of pollen fertility, *i*.*e*., 75.67% and 66.70%, respectively, as compared with control (92.10%) (**Figures 3A, B**).

### Chlorophyll and carotenoid content

The chlorophyll and carotenoid contents were significantly affected by the treatment of caffeine and SA. The results showed that chlorophyll developed more (*i*.*e*., 3.15 and 2.98 mg/g) at the lowest concentration (0.2%) of caffeine and SA, respectively, as compared with the control (2.67 mg/g), as illustrated in **Figures 3C, D**. The carotenoids likewise showed an increase over the control. The highest carotenoid levels with 1.02 and 0.87 mg/g were observed at 0.4% and 0.2% treatments of caffeine and SA, respectively. The highest dose of mutagen led to a decline in the chlorophyll and carotenoid contents compared with untreated plants (**Figures 3C, D**).

### Proline content

In this experiment, the impact of different treatments of caffeine and SA, respectively, have been displayed on proline content compared with the control ones. A significant increase was also observed in caffeine and SA treatments. It was depicted that the proline content of the control plant is 17.63 µg/g FW, which significantly increases to 46.50 and 47.20 µg/g FW in a 1.0% concentration of caffeine and SA. A continuous increase in proline content was detected with increasing doses of caffeine and SA (**Figures 3C, D**).

### Quantitative traits

The observations recorded on different agronomic traits in caffeine and SA treatments are illustrated in **Table 1**. The shoot length, root length, pods bearing branches/plant, number of pods/plant, number of seeds/pod, 1,000 seed weight (g), and total yield (g) exhibited a reduction at higher doses of the mutagen. Exposing seeds to lower concentrations (0.2% and 0.4%) of mutagen caused enhancement in the quantitative parameters of the treated plants against control. The average shoot length of the control population was 66.70 cm; it increased significantly up to 73.20 and 71.80 cm at 0.2% of caffeine and SA, respectively. However, it decreased at higher doses of the mutagen as compared with the control. The root length of the control was 19.80 cm and increased to 22.50 cm (0.2%) in the caffeine treatments, while in the SA treatments, it gets improved to 21.40 cm (0.2% SA). The average pods bearing branches per plant in control was 3.20; it remarkably increased to 4.20 and 3.65 in 0.2% of caffeine and SA, respectively, and decreased at higher doses (1.0%) of the mutagen. There was a remarkable positive change in the average number of pods per plant in the lower concentration of the mutagen. In the rest of the treatments, it decreases over the control. The number of seeds per pod was 9.60 in the control plants, while the maximum seeds recorded were 11.00 and 10.40 with 0.4% and 0.2% concentration of caffeine and SA, respectively. The 1,000 seed weight was significantly increased in 0.2% treatment of both mutagens. The total yield per plant is higher in caffeine-treated plants, with 2.56 g in 0.2% concentration.

**Table 1:** Effect of caffeine and sodium azide (SA) on the quantitative traits of *Trigonella foenum-graecum* L. (M_1_ generation).

Concentration	Shoot length (cm)	Root length (cm)	Fertile branches per plant	Pods per plant	Seeds per pod	1,000 seed weight	Seed yield
(%)	(mean ± SE)	(mean ± SE)	(mean ± SE)	(mean ± SE)	(mean ± SE)	(g)	(g)
						(mean ± SE)	(mean ± SE)
Control	66.70 ± 1.008^d^	19.80 ± 0.876^b^	3.20 ± 0.132^de^	15.20 ± 0.479^abc^	9.60 ± 0.261^ef^	11.45 ± 0.293^bcde^	1.82 ± 0.151^bcde^
Caffeine							
0.2	73.20 ± 1.014^a^	22.50 ± 0.935^a^	4.20 ± 0.131^a^	16.00 ± 0.354^a^	10.60 ± 0.179^ab^	12.53 ± 0.250^a^	2.56 ± 0.176^a^
0.4	71.40 ± 1.030^ab^	21.10 ± 1.197^ab^	3.80 ± 0.092^b^	15.80 ± 0.343^ab^	11.00 ± 0.162^a^	12.15 ± 0.244^ab^	2.29 ± 0.180^ab^
0.6	69.60 ± 1.266^bcd^	20.40 ± 1.064^ab^	3.40 ± 0.103^cd^	15.40 ± 0.390^abc^	10.20 ± 0.188^bcd^	11.88 ± 0.191^abc^	2.01 ± 0.184^bcd^
0.8	61.10 ± 2.984^e^	17.30 ± 0.662^c^	3.00 ± 0.101^ef^	14.80 ± 0.293^bcd^	9.40 ± 0.141^f^	11.40 ± 0.141^cde^	1.62 ± 0.149^def^
1	55.40 ± 0.974^f^	15.40 ± 0.671^cd^	2.60 ± 0.075^gh^	14.40 ± 0.316^cd^	8.80 ± 0.228^g^	10.83 ± 0.233^e^	1.32 ± 0.128^f^
SA							
0.2	71.80 ± 0.901^ab^	21.40 ± 0.271^ab^	3.65 ± 0.077^bc^	15.80 ± 0.190^ab^	10.40 ± 0.027^bc^	12.46 ± 0.299^a^	2.29 ± 0.030^ab^
0.4	70.20 ± 0.565^abc^	20.60 ± 0.160^ab^	3.44 ± 0.073^cd^	15.40 ± 0.172^abc^	10.00 ± 0.047^cde^	12.04 ± 0.122^abc^	2.12 ± 0.039^abc^
0.6	67.50 ± 0.451^cd^	19.90 ± 0.204^b^	3.30 ± 0.058^de^	15.25 ± 0.178^abc^	9.80 ± 0.029^def^	11.62 ± 0.102^bcd^	1.97 ± 0.032^bcd^
0.8	59.40 ± 0.501^e^	15.60 ± 0.194^c^	2.80 ± 0.077^fg^	14.60 ± 0.155^cd^	8.80 ± 0.079^g^	10.95 ± 0.089^de^	1.72 ± 0.027^cdef^
1	52.20 ± 0.569^g^	13.20 ± 0.223^d^	2.40 ± 0.057^h^	14.00 ± 0.089^d^	8.20 ± 0.055^h^	10.76 ± 0.032^e^	1.44 ± 0.018^ef^

Mean within columns followed by the same letter is not significant at 0.05% level of significance based on Duncan Multiple-Range Test.

SE, standard error.

### Morphological variants/mutants in M_1_ and M_2_ generation

**Figure 4:**
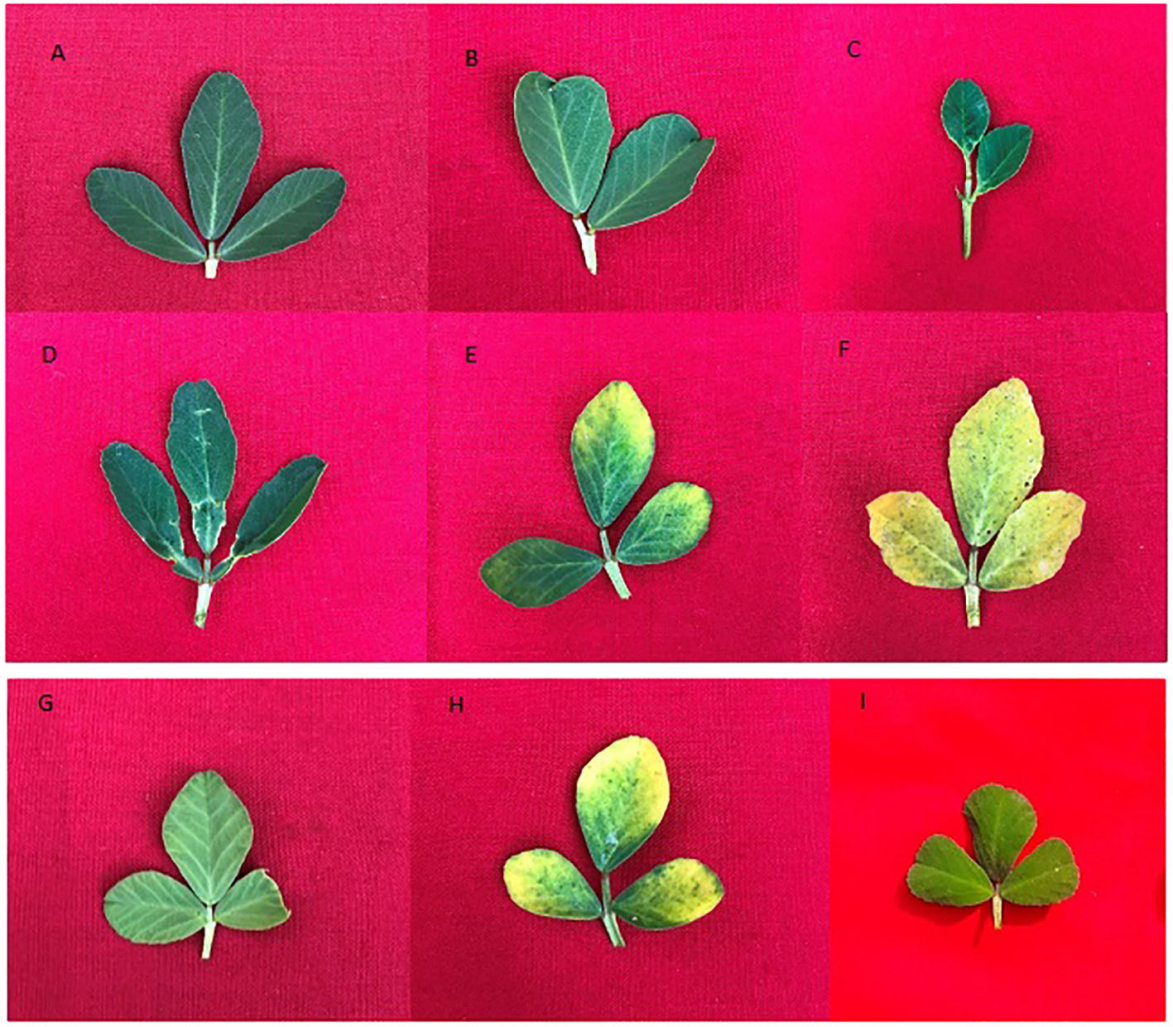
**(A)** Control leaf, **(B–F)** morphological and chlorophyll variants of M1 generation and **(G–I)** morphological and chlorophyll mutants of M_2_ generation.

**Figure 5:**
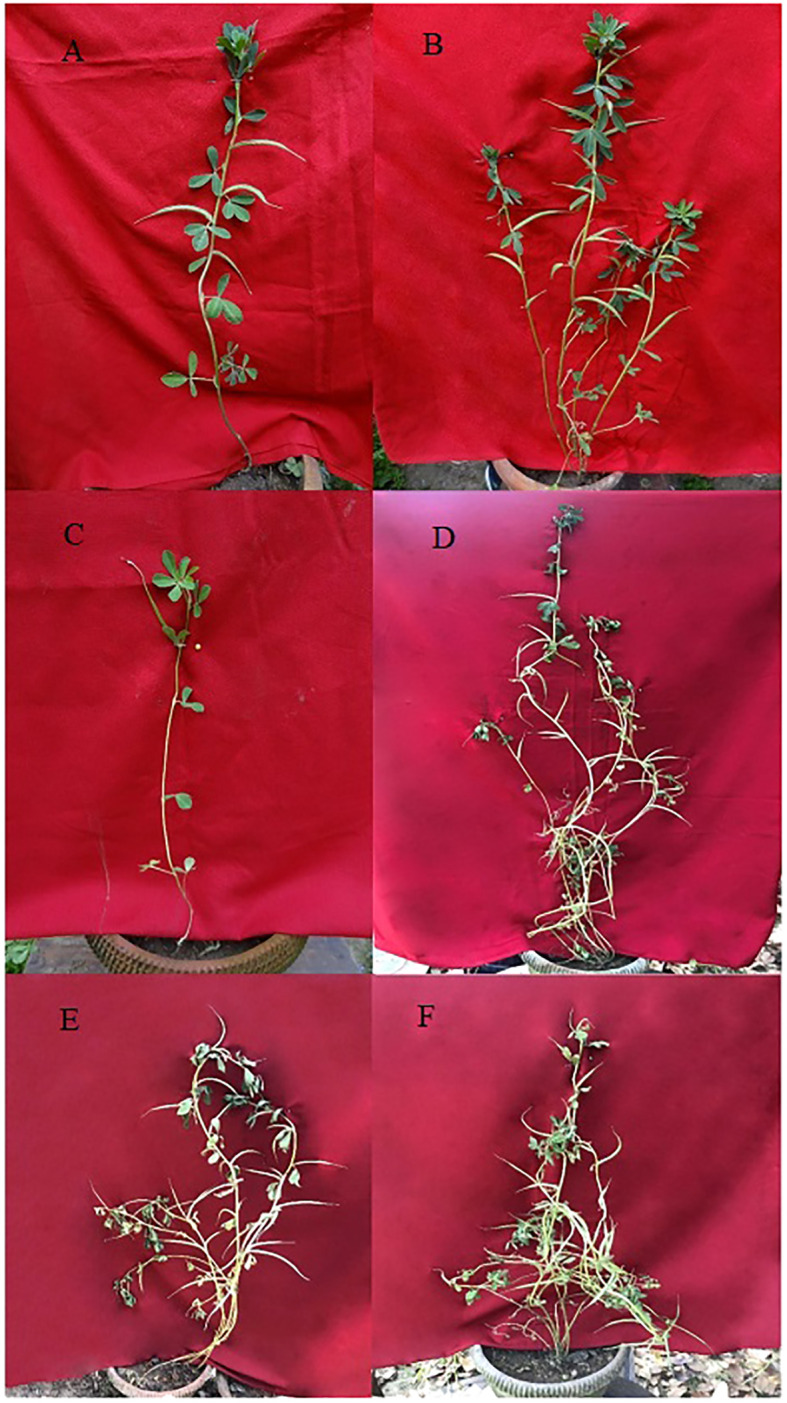
Variations and mutations observed in the plant size and plant growth habit during the M_1_ and M_2_ generation. **(A)** Control, **(B)** Tall plant, **(C)** Dwarf plant, **(D)** Tall mutant, **(E)** High yielding mutant and **(F)** Bushy mutant.

**Figure 6:**
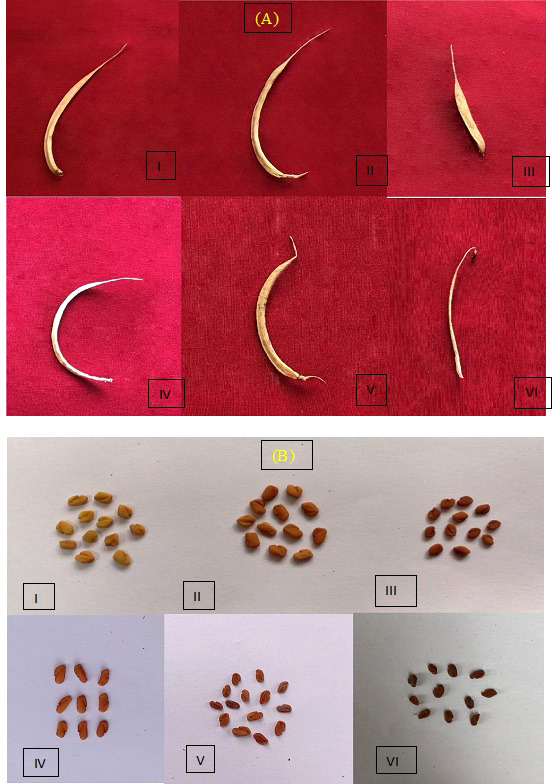
**(A)** Morphological mutations (altered pod shape and size) observed during the M_2_ generation. **(B)** Morphological mutations (altered seed size, shape, and color) observed during M_2_ generation.

A broad spectrum of mutagen-induced phenotypic variations/mutations was observed in the M_1_ and M_2_ populations. Several variations are beneficial from a yield improvement perspective in fenugreek. These variations include plant height (tall and dwarf), growth habit (bushy), leaf, pod, and seed recorded. The control vegetative leaf had normal, dark green, entire, opposite, three small obovate to oblong leaflets (**Figure 4A**). The treated plants showed vegetative leaf variants in M_1_, including broad deformed bifoliate leaflets, which were deeply notched at the apex (at 0.2% caffeine, **Figure 4B**), two small, ovate leaflets with one rudimentary leaflet (at 0.4% SA, **Figure 4C**), leaflets that were deeply notched on both sides near the base with a wavy margin (at 0.6% caffeine, **Figure 4D**), two leaflets with chlorophyll mutations at the apices (at 0.6% SA, **Figure 4E**), and all leaflets with xantha chlorophyll mutations (at 0.8% SA, **Figure 4F**). In the M_2_ generation, few vegetative leaf mutants were observed, such as unequal-sized leaflets, one obovate, second ovate, and third leaflet with undeveloped tip (at 0.4% caffeine, **Figure 4G**), chlorophyll mutant with marginal pigmentation (at 0.6% SA, **Figure 4H**), and small-sized leaflets (at 1.0% SA, **Figure 4I**). Compared with the control, several variants/mutants with altered plant height and growth habits were observed in caffeine and SA. The control plant had moderate height and normal branching with an average yield (**Figure 5A**). At the same time, the variants include tall height, with more branches and increased yield (at 0.2% caffeine, **Figure 5B**) and a dwarf plant with a single pod and low yield (at 0.8% SA, **Figure 5C**). In the M_2_ population, it includes a tall mutant with more number of branches and normal yield (at 0.2% caffeine, **Figure 5D**), a mutant with normal height and high yield (at 0.4% caffeine, **Figure 5E**), and a bushy mutant with an increased number of branches and leaves and high yield (at 0.2% SA, **Figure 5F**). The M_2_ population exhibited an alteration in the shape and size of the pods, *i*.*e*., long pod (0.4% caffeine, **Figure 6A-II**), small (1.0% caffeine, **Figure 6A-III**), curved (0.2% SA, **Figure 6A-IV**), bold-seeded pod (0.4% SA, **Figure 6A-V**), and narrow pod (1.0% SA, **Figure 6-AVI**). Moreover, the control seeds are smooth and rhomboidal with a deep, oblique furrow on the side, yellowish-brown in color (**Figure 6B-I**). In comparison, the treatment of caffeine and SA showed a higher number of seed mutants in M_2_ generation, such as bold seeds (0.2% caffeine, **Figure 6B-II**), small-sized and dark-colored seeds (0.2% SA, **Figure 6B-III**), elongated seeds (0.4% caffeine, **Figure 6B-IV**), wrinkled surfaced seeds (0.8% caffeine, **Figure 6B-V**), and dark-colored and wrinkled seeds (1.0% SA, **Figure 6B-VI**). The beneficial mutations were produced at lower and intermediate treatments of the caffeine and SA in fenugreek.

#### Meiotic abnormalities

**Figure 7:**
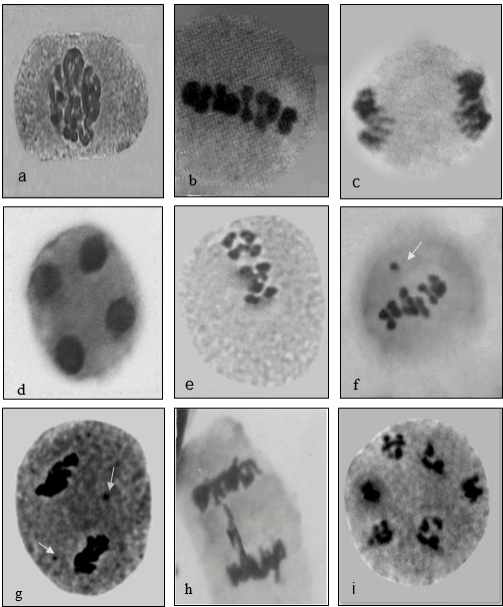
Caffeine and sodium azide-induced chromosomal abnormalities in *Trigonella.* **(A)** Diakinesis, **(B)** metaphase, **(C)** anaphase, **(D)** telophase-II, **(E)** disoriented metaphase-I, **(F)** laggard, **(G)** stickiness, **(H)** chromatin bridge, and **(I)** multinucleate.

Fenugreek is a diploid with chromosome number 2n=16, with 8 bivalents that are smaller in size. Meiotic analysis of the plants upraised from the mutagen-treated seeds showed a high frequency of chromosomal anomalies such as disturbed polarity, laggards, univalents, bridges, stickiness, etc., at different stages of meiosis. Normal meiosis was observed in control, displaying 8 bivalents in the diakinesis stage organized typically at metaphase-I followed by segregating into 8:8 in anaphase I observed in the pollen mother cells (PMCs) of floral buds of untreated plants. The diakinesis showed 8 bivalents arranged in a ring (Control, **Figure 7A**), chromosomes arranged at the equator (Control, **Figure 7B**), chromosomes located at the opposite poles anaphase-I (Control, **Figure 7C**), telophase II exhibited four clusters of chromosomes present at the respective poles (Control, **Figure 7D**), disturbed orientation at metaphase-I (0.4% caffeine, **Figure 7E**), metaphase-I with one laggard (0.6% SA, **Figure 7F**), stickiness with two laggards (0.8% caffeine, **Figure 7G**), chromatin bridge at anaphase-I (0.8% SA, **Figure 7H**), and multinucleate condition (1.0% SA, **Figure 7I**). The maximum variation frequency in the chromosomal structure was observed in SA as compared with caffeine reflecting different sensitivities towards mutagenic treatments (**Table 2**).

**Table 2:** Frequency of chromosomal abnormalities induced by caffeine and sodium azide (SA) in *Trigonella foenum graecum* L. (M_1_ generation).

		Prophase-I (diakinesis)			Metaphase-I/II							Anaphase-I/II				Telophase-I/II										
Conc .of mutagens (%)	Total number of pollen mother cells (PMCs) observed	Univalents	Multivalents	% of abnormal PMCs		Univalents	Multivalents	Precocious Mov	Stray chromosome chromosomes	Stickiness	% of abnormal PMCs	Laggards	Bridges	Unequal sep.	% of abnormal PMCs	Laggards	Bridges	Unequal sep.	Micronucleate	Multinucleate	Disturbed polarity	Cytomixis	% of abnormal PMCs		Total number of abnormal PMCs observed	Total % of abnormal PMCs observed
Control	263	-	-	-		-	-	-	-	-	-	-	-	-	-	-	-	-	-	-	-	-	-		-	-
Caffeine (%)																										
0.2	221	-	-	0.00		-	1	-	-	1	0.90	1	-	-	0.45	1	-	1	-	-	1	-	1.36	6		**2.71**
0.4	228	1	-	0.44		1	1	-	-	1	1.32	1	1	-	0.88	1	1	1	1	-	1	1	2.63	12		**5.26**
0.6	237	-	1	0.42		1	2	1	1	2	2.95	2	1	1	1.69	2	1	2	1	1	2	1	4.22	22		**9.28**
0.8	256	2	2	1.56		2	2	1	2	3	3.91	2	2	1	1.95	2	2	2	2	2	3	2	5.86	34		**13.28**
1.0	271	2	3	1.85		3	3	2	3	4	5.54	2	2	2	2.21	3	3	3	3	3	4	3	8.12	48		**17.71**
SA (%)																										
0.2	231	1	-	0.43		1	1	-	1	1	1.73	1	-	1	0.87	1	-	2	1	-	1	1	2.60	13		**5.63**
0.4	248	1	1	0.81		-	1	-	2	2	2.02	2	-	1	1.21	1	2	1	2	-	2	1	3.63	19		**7.66**
0.6	265	1	2	1.13		2	2	1	3	2	3.77	3	2	1	2.26	2	2	2	3	3	2	2	6.04	35		**13.21**
0.8	279	3	3	2.15		3	3	2	3	3	5.02	3	2	2	2.51	4	4	2	3	3	4	4	8.60	51		**18.28**
1.0	294	4	5	3.06		3	4	4	5	5	7.14	3	3	4	3.40	4	4	4	3	4	5	4	9.52	68		**23.13**

#### Total protein content, phenolic content, and flavonoid content

The maximum increase in protein content (51.65 and 50.21 mg/g FW) in response to treatments of caffeine and SA was obtained at 0.6% concentration as compared with the control. The protein content of the control plant is 39.42 mg/g FW. In contrast, minimum protein content was recorded at higher doses (**Table 3**). Based on the present findings, both mutagen 0.6% and 1.0% concentrations triggered a remarkable increase and decrease in phenolic content. The highest phenolic content was 195.74 mg gallic acid/g FW in caffeine and 199.4 mg gallic acid/g FW in SA treatments over the control with 165.32 mg gallic acid/g FW (**Table 3**). Mutagen impact on plant significantly increased the flavonoid content to 35.14 mg quercetin/g FW and 37.90 mg quercetin/g FW in 0.6% concentration from their control with 24.36 mg quercetin/g FW (**Table 3**). A higher (1.0%) concentration of caffeine and SA shows high toxicity.

**Table 3:** Impact of caffeine and sodium azide (S)A on total protein content, total phenolic content, and total flavonoids of *Trigonella foenum*-*graecum* L. (M_1_ generation).

Concentration	Total protein content	Phenolic	Flavonoids
(%)	(mg/g FW)	(mg gallic acid/g FW)	(mg quercetin/g FW)
	(mean ± SE)	(mean ± SE)	(mean ± SE)
Control	39.42 ± 2.426^cde^	165.32 ± 3.129^e^	24.36 ± 1.870^def^
Caffeine			
0.2	45.37 ± 2.650^abcd^	182.50 ± 3.106^cd^	27.45 ± 1.850^cde^
0.4	48.25 ± 2.946^abc^	189.45 ± 2.836^bc^	32.56 ± 1.761^abc^
0.6	51.65 ± 2.700^a^	195.74 ± 2.558^ab^	35.14 ± 2.130^ab^
0.8	42.15 ± 2.613^bcde^	178.25 ± 2.865^d^	26.78 ± 1.718^cdef^
1	36.54 ± 2.520^de^	161.67 ± 2.297^e^	21.36 ± 1.216^f^
SA			
0.2	43.68 ± 2.348^abcd^	183.90 ± 2.233^cd^	29.50 ± 1.587^bcd^
0.4	47.56 ± 2.371^abc^	192.50 ± 2.204^ab^	34.80 ± 1.725^ab^
0.6	50.21 ± 2.537^ab^	199.40 ± 1.808^a^	37.90 ± 1.577^a^
0.8	41.12 ± 2.362^bcde^	180.60 ± 2.007^d^	28.70 ± 1.548^cd^
1	34.36 ± 2.275^e^	160.90 ± 2.193^e^	22.40 ± 1.042^ef^

Mean within columns followed by the same letter is not significant at 0.05% level of significance based on Duncan Multiple-Range Test.

SE, standard error.

### M_2_ generation observations

#### Quantitative traits

The quantitative traits such as shoot length (cm), root length (cm), number of fertile branches per plant, number of pods per plant, number of seeds per pod, 1,000 seed weight (g), and seed yield (g) followed a similar trend as in M_1_ generation. The values of quantitative traits were comparatively higher than the M_1_ generation at their respective concentrations (**Table 4**).

**Table 4:** Effect of caffeine and sodium azide (SA) on the quantitative traits of the treated population of *Trigonella foenum-graecum* L. (M_2_ generation).

Concentration (%)	Shoot length (cm) (mean ± SE)	Root length (cm) (mean ± SE)	Fertile branches per plant (mean ± SE)	Pods per plant (mean ± SE)	Seeds per pod (mean ± SE)	1,000 seed weight (g) (mean ± SE)	Seed yield (g) (mean ± SE)
Control	67.10 ± 0.498^c^	20.7 ± 0.418^b^	3.40 ± 0.141^bcde^	15.40 ± 0.130^bcde^	9.80 ± 0.105^bcd^	11.48 ± 0.226^bcd^	1.84 ± 0.127^cd^
Caffeine							
0.2	73.90 ± 0.598^a^	23.80 ± 0.475^a^	4.40 ± 0.170^a^	16.20 ± 0.155^a^	10.90 ± 0.186^a^	12.55 ± 0.248^a^	2.59 ± 0.141^a^
0.4	72.60 ± 0.669^ab^	22.40 ± 0.493^ab^	4.00 ± 0.192^ab^	16.00 ± 0.212^ab^	10.60 ± 0.203^ab^	12.18 ± 0.262^ab^	2.32 ± 0.150^ab^
0.6	70.50 ± 0.702^b^	21.70 ± 0.680^b^	3.80 ± 0.221^abc^	15.80 ± 0.230^abc^	10.40 ± 0.248^abc^	11.91 ± 0.271^abc^	2.03 ± 0.163^bc^
0.8	62.90 ± 0.729^d^	17.90 ± 0.871^c^	3.20 ± 0.228^cdef^	15.00 ± 0.277^def^	9.50 ± 0.262^cd^	11.42 ± 0.284^bcd^	1.78 ± 0.172^cd^
1.0	57.50 ± 0.758^f^	16.10 ± 1.023^cd^	2.80 ± 0.261^ef^	14.80 ± 0.326^ef^	8.90 ± 0.280^de^	10.85 ± 0.293^d^	1.49 ± 0.186^d^
SA							
0.2	72.60 ± 0.698^ab^	22.10 ± 0.483^ab^	3.80 ± 0.212^abc^	16.00 ± 0.152^ab^	10.60 ± 0.350^ab^	12.50 ± 0.253^a^	2.38 ± 0.154^ab^
0.4	70.90 ± 0.797^b^	21.50 ± 0.488^b^	3.60 ± 0.220^bcd^	15.60 ± 0.219^abcd^	10.20 ± 0.447^abc^	12.09 ± 0.262^ab^	2.22 ± 0.163^abc^
0.6	68.30 ± 0.830^c^	20.80 ± 0.616^b^	3.40 ± 0.235^bcde^	15.20 ± 0.249^cde^	9.90 ± 0.556^abcd^	11.78 ± 0.280^abc^	1.99 ± 0.172^bc^
0.8	60.40 ± 0.863^e^	16.30 ± 0.636^cd^	3.00 ± 0.250^def^	14.80 ± 0.221^ef^	9.10 ± 0.476^de^	11.21 ± 0.297^cd^	1.76 ± 0.181^cd^
1.0	53.10 ± 0.941^g^	14.50 ± 0.672^d^	2.60 ± 0.266^f^	14.40 ± 0.303^f^	8.40 ± 0.571^e^	10.79 ± 0.306^d^	1.47 ± 0.199^d^

SE, standard error.

*Mean within columns followed by the same letter is not significant at 0.05% level of significance based on Duncan Multiple Range Test.

#### Mutagenic effectiveness and efficiency

The mutagenic effectiveness and efficiency of various mutagens on fenugreek cultivar were varied (**Table 5**). The results revealed that effectiveness and efficiency were maximum at lower concentrations of the mutagen treatments. Mutagenic effectiveness was observed to be highest in SA, followed by caffeine. It decreased from 3.38 to 2.33 and 5.48 to 2.68 in 0.2%–1.0% caffeine and SA, respectively. Mutagenic efficiency has been worked out based on the extent of biological damage, *i*.*e*., chromosomal abnormalities (Mp/Me). It was found to have decreased from 2.25 to 1.18 and 1.75 to 1.04 in 0.2%–1.0% caffeine and SA, respectively (**Table 5**).

**Table 5:** The number of M_1_ plant progenies, progenies segregating in M_2_ population, mutated plant percent, mutagenic effectiveness, and efficiency induced by caffeine and sodium azide (SA).

Mutagen treatment	Number of seeds sown in M_1_	Number of seeds germinated in M_1_	Number of fertile M_1_ plant progenies	Number of seeds sown in M_2_	Number of seeds germinated in M_2_	Number of fertile M_2_ plants	Number of M_2_ mutants	Mutated plant (%) Mp	Biological damage in M_1_ generation (% meiotic abnormalities) Me	Mutagenic effectiveness	Mutagenic efficiency (Mp/Me)
Control	100	91	89	100	92	91	–		–	–	–
Caffeine (%)											
0.2	100	85	82	100	87	85	5	6.09	2.71	3.38	2.25
0.4	100	82	80	100	84	83	9	11.25	5.26	3.13	2.14
0.6	100	76	74	100	78	75	10	13.51	9.28	2.50	1.45
0.8	100	71	68	100	74	70	12	17.65	13.28	2.45	1.33
1.0	100	65	62	100	68	64	13	20.97	17.71	2.33	1.18
SA (%)											
0.2	100	83	81	100	85	83	8	9.87	5.63	5.48	1.75
0.4	100	79	77	100	81	78	9	11.68	7.66	3.24	1.52
0.6	100	72	69	100	75	72	11	15.94	13.21	2.95	1.21
0.8	100	66	64	100	67	65	12	18.75	18.28	2.60	1.03
1.0	100	61	58	100	63	60	14	24.14	23.13	2.68	1.04

#### Stomatal behaviour of leaf and micromorphology of seeds

**Figure 8:**
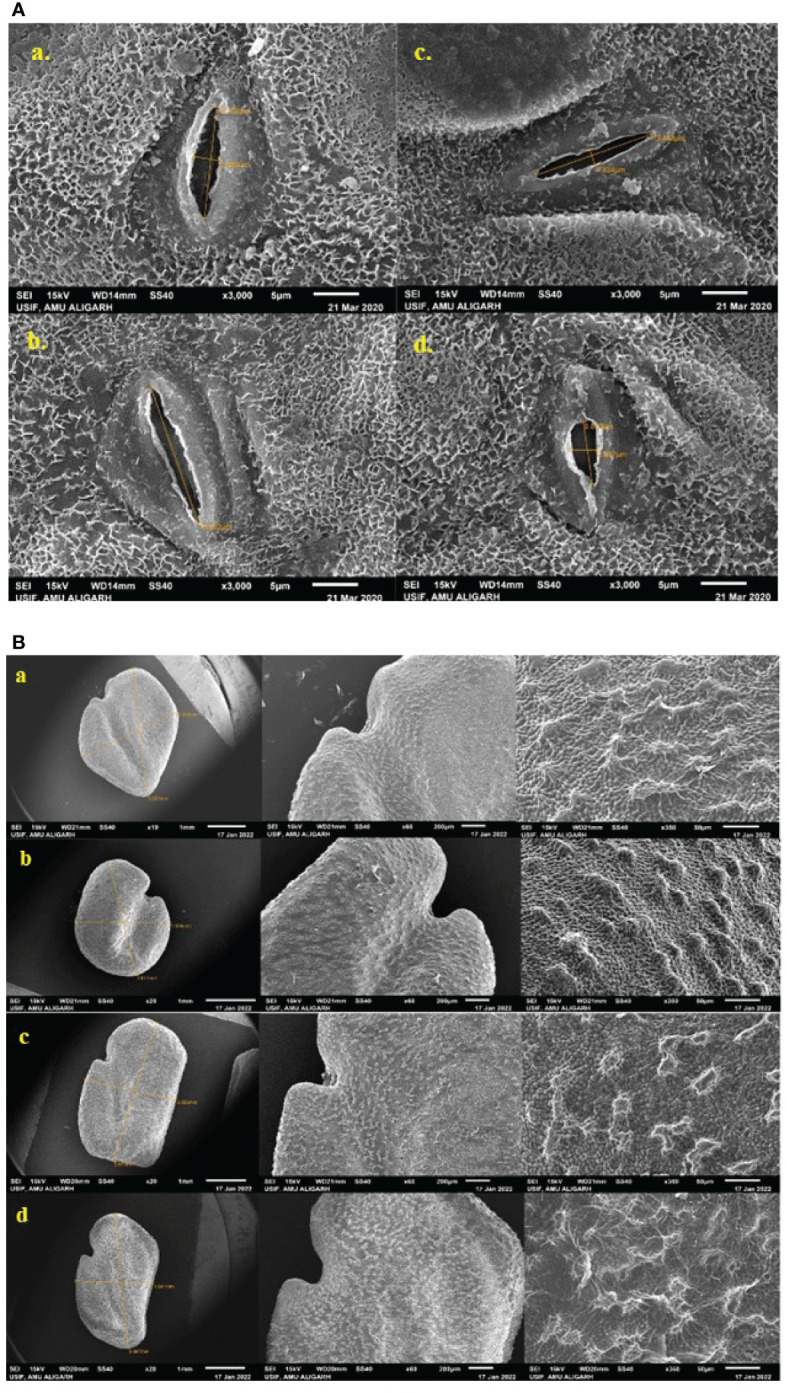
**(A)** Changes in the size of stomatal aperture of the mutant leaf and **(B)** micromorphology of the mutant seeds under a scanning electron microscope.

In this analysis, SA has more harmful results on stomata than caffeine; the stomatal aperture size decreases as the concentration of the mutagen increases or may be completely closed (**Figure 8A**). The control plant has a stomatal aperture of size 12.389 μm in length and 2.839 μm in width (**Figure 8Aa**). M2 plant progenies have varying stomatal length and width, such as in 0.2% caffeine, the length of stomata was 15.802 μm (**Figure 8Ab**), the stomatal size was 13.453 μm and 1.834 μm in 0.2% SA treatment (**Figure 8Ac**), and in 0.8% SA, the stomatal size was 6.873 μm in length and 2.667 μm in width (**Figure 8Ad**). The SEM microphotographs showed the variations in shape, size, and surface of fenugreek seeds (**Figure 8B**). The M2 generation seeds are of irregular shape, size, and surface includes, the control seed is rhomboidal in shape, measuring 3.557 mm & 2.806 mm in length and width with a slightly lateral and oblique groove (**Figure 8Ba**), a small oval-shaped seed with a prominent furrow has 2.611 mm in length and 1.904 mm in width (0.2% SA, **Figure 8Bb**), elongated (oblong) seed with irregular shape (3.071 mm×2.353 mm) (0.4% caffeine, **Figure 8Bc**) and regular size seed with wrinkled texture (3.487 mm×1.941 mm) was also observed (0.8% caffeine, **Figure 8Bd**).

#### Frequency of M_2_ morphological mutation

**Figure 9:**
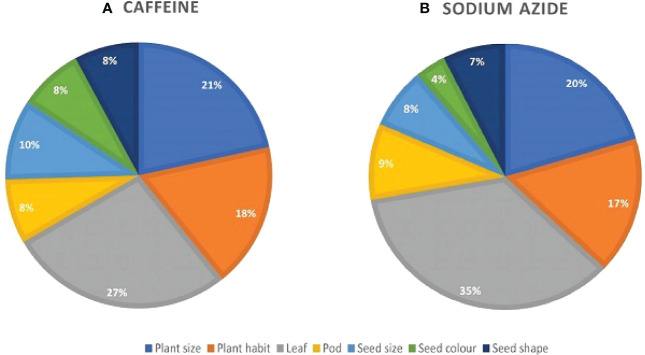
Comparative study of phenotypic mutants by seven major phenotypic categories in M_2_ plants of mutagenized population: **(A)** caffeine-treated populations. **(B)** Sodium azide-treated populations.

**Figure 10:**
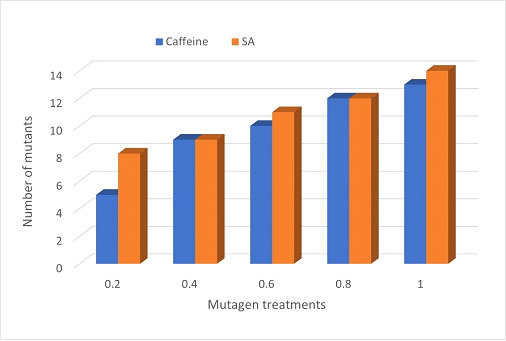
Comparative illustration of M_2_ mutants induced by caffeine and sodium azide.

A broad spectrum of mutant phenotypes was developed in the M_2_ population; several of them were found to be viable based on their quantitative traits. Various morphological mutants with modified characters affecting plant height, growth habit, leaf, pod, seed size, seed shape, and seed color were observed at different concentrations in M_2_ generation. The spectrum of mutations was wider in SA than in caffeine (**Figure 8**), as caffeine produced 51 while SA produced 54 individual mutants under seven major categories (**Table 6**). The maximum frequency of morphological mutants was observed in 1.0% SA (24.14) than 1.0% caffeine (20.97) (**Table 5**). The characterization of the phenotypic categories and the frequencies for both mutagens are shown in **Table 6**, while the frequencies of the mutation in each morphological category out of the total morphological mutation are demonstrated in **Figure 9**. In caffeine, leaf mutants were the highest (27%), followed by plant size (21%), plant habit (18%), seed size (10%), pod (8%), seed color (8%), and seed shape mutants (8%) (**Figure 9A**), while in SA leaf mutants were the highest (35%), followed by plant size (21%), plant habit (18%), pod (9%), seed size (8%), seed shape (7%), and seed color mutants (4%) (**Figure 9B**). Overall, the maximum frequency of morphological mutants was associated with leaf, followed by plant size, plant growth habit, pod, seed size, seed shape, and seed color (**Figure 10**).

**Table 6:** Frequency and spectrum of the phenotypic characters of the observed mutants during the M_2_ generation.

Mutants		Caffeine		Sodium azide		Total		Grand total	
		Number of mutants	Frequency (%)	Number of mutants	Frequency (%)	Number of mutants	Frequency (%)	Number of mutants	Frequency (%)
Plant size	Tall	7	0.14	6	0.11	13	0.12	22	0.21
	Dwarf	4	0.08	5	0.09	9	0.09		
Plant habit	Bushy	4	0.08	3	0.05	7	0.07	18	0.18
	Unbranched	2	0.04	3	0.05	5	0.05		
	Few branches	3	0.06	3	0.05	6	0.06		
Leaf	Leaf shape	6	0.12	8	0.15	14	0.13	33	0.31
	Leaf size	6	0.12	7	0.13	13	0.12		
	Other leaf morphology	2	0.04	4	0.07	6	0.06		
Pod	Small/narrow	1	0.02	2	0.04	3	0.03	9	0.09
	Bold seeded pod	2	0.04	2	0.04	4	0.04		
	Curved	1	0.02	1	0.02	2	0.02		
Seed size	Small	2	0.04	3	0.05	5	0.05	9	0.09
	Large	3	0.06	1	0.02	4	0.04		
Seed color	Yellowish brown	2	0.04	1	0.02	3	0.03	6	0.06
	Brown	2	0.04	1	0.02	3	0.03		
Seed shape	Bold smooth	3	0.06	1	0.02	4	0.04	8	0.08
	Elongated smooth	0	0.00	1	0.02	1	0.01		
	Wrinkled	1	0.02	2	0.04	3	0.03		
Total		51	1.02	54	0.99	105	1.02		

## Discussion

These present findings revealed that the treatment of chemical mutagens stimulated the diversity in morpho-physiological traits, quality, and yield of the plant (Siddiqui et al., 2007; Samatadze et al., 2019). Chemical mutagens were shown to influence biological changes in the population through DNA base substitution (Cooper et al., 2008).

In the present study, high doses of caffeine and SA treatments exhibited a severe reduction in biological parameters of the cultivar. Seed germination is one of the significant parameters for finding the mutagen effect on plants. Suppression in germination rate may be attributed to metabolic disruption (Ananthaswamy et al., 1971) or chromosomal deletion (Khan et al., 2007). Moreover, azide anions are potent proton pump inhibitors (Kleinhofs et al., 1978) altering the mitochondrial membrane potential (Zhang et al., 2000), resulting in a shortage of ATP and slowing down the germination. This result is consistent with earlier studies on several crops, including *Cajanus cajan* (Saroj et al., 2016), *Coriandrum sativum* (Kolhe et al., 2020), *Trigonella foenum-graecum* (Hassan et al., 2018; Naaz et al., 2020), and *Lens culinaris* (Sharma et al., 2022). The survival at maturity was reduced with an increasing concentration of mutagens due to cytogenetic aberrations and physiological instabilities (Sato and Gaul, 1967) and disruption in the stability among inhibitors of growth regulators and promoters (Meherchandani, 1975). Similar results were observed by Naaz et al. (2020) in *Trigonella foenum-graecum*, Aslam et al. (2017) in *Capsicum annum*, Tantray et al. (2017) in *Nigella sativa*, and Shahwar et al. (2020) in *Lens culinaris.* Here a drop in pollen fertility percentage has been observed after mutagen treatments and earlier reported in mungbean (Veni et al., 2017), fenugreek (Choudhary et al., 2012), and *Linum usitatissimum* (Jahan et al., 2020). Kumar and Rai (2007) observed that higher chromosomal anomalies greatly influenced microsporogenesis, which is responsible for the development of non-viable gametes, thus significantly reducing pollen fertility.

The critical prerequisite to set off breeding techniques is the ample accessibility of genetic variability in the crop gene pool that defines the success of trait improvement. A remarkable rise in the quantitative characters, including shoot length, root length, pods bearing branches/plant, number of pods/plant, number of seeds/pod, and yield was detected at lower and intermediate concentrations of the mutagen against the control. It showed an inhibitory effect at higher doses of the mutagen. Reduced plant height has been described earlier in several crops such as *Trigonella foenum-graecum* (Hassan et al., 2018), *Lens culinaris* (Durre et al., 2018), *Capsicum annum* (Hasan et al., 2022b). The reduction in plant height may be due to changes in ascorbic acid and physiological disturbances (Dhamayanthi and Reddy, 2000). Furthermore, some pleiotropic genes may regulate plant height (Wei et al., 2010; Yano et al., 2016).

The mean number of branches at lower concentrations increased significantly in the M_2_ generation. These results align with earlier reports (Hassan et al., 2018; Naaz et al., 2020) in fenugreek and (Goyal et al., 2022) in urdbean. The increased fertile branches per plant in lower-dose mutagen-treated populations may be attributed to the reduced synthesis of strigolactones. Many workers have reported a significant increase in pod per plant in crops such as *Nigella sativa* (Amin et al., 2019) and *Sesamum indicum* (Pradhan and Paul, 2019). The mean number of seeds per pod as well as the total seed yield per plant are interrelated with one another. The seeds per pod exhibited the stability of the parameter after mutagen treatments, but the total seeds per plant improved remarkably in the lower and intermediate doses of caffeine and SA. In a breeding program, yield is considered a reliable parameter, and a plant breeder ultimately intends to enhance the yield and its related characteristics. In grain legumes, seed yield is controlled by many other quantitative features, namely, pods bearing branches, pods per plant, seeds per pod, seed weight, and, eventually, total seed yield per plant. These traits are directly correlated to seed yield. The M_1_ and M_2_ generation results revealed that higher doses exhibited inhibitory effects on yield traits, while lower doses showed stimulatory effects on yield-attributing characters. These results agreed with previous studies (Verma et al., 2017; Hanafy and Akladious, 2018; Naaz et al., 2020; ALshamrani et al., 2022). The diminution in quantitative traits at higher concentrations may arise due to physiological disturbance or DNA damage triggered in the plant cells by the mutagen (Thilagavathi and Mullainathan, 2011). In this study, these observations were supported by Amin et al. (2015) and Sharma et al. (2022) in lentil and Hasan et al. (2020) in chili.

Chromosomal abnormalities during meiosis due to mutagens are regarded as the most dependable indices to assess the effectiveness of the mutagen. The chromosome stickiness arises due to histone protein malfunctioning, which leads to the improper folding of DNA (Myers et al., 1992; Kumar and Rai, 2007), and is the most common type of meiotic abnormalities. The presence of chromatin bridges at anaphase-I/II might be associated with the stickiness of chromosome ends or breakage or reunion of chromosomes, which is observed in *Trigonella foenum-graecum* (Choudhary et al., 2012) and *Nigella sativa* (Amin et al., 2015).

Several workers have reported the occurrence of chlorophyll mutation in *Trigonella* (Hassan et al., 2018; Kavina et al., 2020; Naaz et al., 2021). The estimated chlorophyll content indicates the photosynthetic efficiency of the crop. Chlorophyll growth appears governed by numerous genes situated on several chromosomes neighboring the centromere and proximal portion of chromosomes (Swaminathan, 1964). Nitrogen is a fundamental structural constituent of chlorophyll and protein molecules; its deficiency disturbs the chloroplast development and chlorophyll accumulation (Demirors Saygideger et al., 2013).

The broad spectrum of morphological variations/mutations of fenugreek, such as altered plant height, growth habit, leaf morphology, and pod and seed shape, denotes the capacity of mutagens to induce genetic variability. The phenotyping of selected mutants was categorized into different categories such as plant size (tall, dwarf), plant habit (bushy, unbranched, branched), leaf (size, shape, number), pod (long, small, curved, bold, narrow), seed size (large, small), color (yellowish-brown, brown), and shape (smooth, wrinkled). The frequency of morphological variations/mutations is attributed to several genes through the pleiotropic effect (Filippetti and De Pace, 1986). It was also observed that the morphological variants were beneficial in mapping studies and in determining the evolution of the crops (Gaur and Gour, 2003). The disparities, such as plant height and habit, leaf morphology, and pod variation, were considered monogenic recessive. Tall and dwarf mutants were observed, which were also earlier observed by Gnanamurthy and Dhanavel (2014) in cowpea and Shahwar et al. (2019) in lentil. The synthesis of phytohormones such as gibberellic acid and brassinosteroids is regulated by many genes, and a mutation in these genes results in altered plant height (Mitchum et al., 2006). Mutations in leaf morphology have also been reported in *Trigonella foenum-graecum* (Choudhary et al., 2012; Hassan et al., 2018; Naaz et al., 2020). The leaf abnormalities are ascribed to chromosomal damages, disturbed enzymatic activities, and accumulation of protein and mineral metabolism (Blixt, 1972; Grover and Virk, 1984). Variations in the seed attributes of mutagenized fenugreek were recorded. Several workers have reported variations in crops such as cowpea (Gnanamurthy and Dhanavel, 2014), black gram (Goyal et al., 2020), and linseed (Jahan et al., 2020). These variations in seeds may be attributed to the mutagen-induced disruption of one or few genes linked with these characteristics.

Proline, an amino acid that accumulates in higher plants, acts in osmo-reductance against environmental stresses and triggers several biological reactions in the cells, leading to various free radicals (Kuznetsov and Shevyakova, 2007; Kumar et al., 2012). The present results were supported by several researchers on multiple crops, such as Hasan et al. (2022b) in *Capsicum annum* and Kumar and Pandey (2019) in *Coriandrum sativum.* Proline content contributes to osmotic adjustment, synthetic reactions of chlorophyll pigments, and inactivation of the Krebs cycle; it scavenges hydroxyl radical as well and is directly involved in stabilizing the structure of macromolecules under stress conditions and indirectly acts as an antioxidant (Mahdavian et al., 2008; Aslam et al., 2014; Theodosiou et al., 2015).

The present work agreed with a similar report of Khater et al. (2016) in *Trigonella foenum-graecum*. The rise in protein content may result from the repression of gene expression, and direct physical effects of chemical mutagenesis led to reduction in the protein content. These observations are revealed by del Carmen Ramıírez-Medeles et al. (2003) and Gorinstein et al. (2001) on *Amaranthus* species.

One of the plants’ most critical secondary metabolites is phenolic compounds, which may directly contribute to their antioxidant activity (Tosun et al., 2009). The present experiment results agreed with several reports, such as in *Trigonella foenum*-*graecum* (Khater et al., 2016; Hanafy and Akladious, 2018). Flavonoids are naturally occurring phenylchromones that function as antioxidants that protect membrane lipids from oxidation (Shahidi et al., 1992; Li et al., 2015). The highest flavonoid content was recorded at lower and intermediate concentrations of the mutagen, and these results are consistent with those of Hanafy and Akladious (2018) in *Trigonella foenum-graecum*. Flavonoids have benefits against cardiovascular disease, age-related degeneration of cell components, and specific forms of cancer (Sulaiman and Balachandran, 2012). The high amount of phenol and flavonoids in fenugreek signifies the higher antioxidant activity of the plants (Noreen et al., 2017).

Mutagenic effectiveness and efficiency are important measures in mutation breeding experiments. Mutagenic effectiveness is defined as the frequency of mutations caused by a unit dose of a mutagen. In contrast, mutagenic efficiency indicates the degree of genetic damage observed in the M_2_ generation relative to the biological damage caused in M_1_ (Konzak et al., 1965). Hence, the response of genotype towards the increasing mutagenic doses is mutagenic effectiveness, and efficiency is the proportion of mutations about the undesirable biological effects. In short, effectiveness signifies genotypic sensitivity, and efficiency reveals mutagenic potency. Here mutagenic effectiveness was the highest in lower concentrations, but it decreased with increasing concentrations. These results are supported by Khan and Tyagi (2010) in soybean, Giri and Apparao (2011) in pigeon pea, Satpute and Fultambkar (2012) in *Glycine max*, Bashir et al. (2013) in *Trigonella foenum-graecum*, and Aslam et al. (2017) in *Capsicum annuum.* A decline in effectiveness at higher doses was observed, indicating that the increase in mutation rate was not proportional to the increase in the doses of the mutagens. The efficiency of mutagenic agent caffeine and SA was determined based on meiotic abnormalities. The present result revealed that lower and intermediate doses were more efficient. Similar findings were observed in *Cicer arietinum* (Mullainathan and Umavathi, 2014), *Vigna umbellata* (Patial et al., 2015), and *Lens culinaris* (Laskar et al., 2017). The higher efficiency of lower and intermediate doses may be ascribed to the progressive increase in biological damage with an increase in dose at a rate higher than the frequency of mutations.

Stomata are the minute openings on the abaxial side of a plant’s leaves. It may be open or close under the control of a pair of kidney-shaped guard cells that perform a very significant function in gaseous exchange. This increased stomatal length and width in the present experiment were earlier reported by Mahadevamma et al. (2012); Mallick et al. (2016), and Qosim et al. (2011). Enhanced stomatal size will increase the transpiration rate and, in turn, lead to increased photosynthesis, thus promoting plant growth and ultimately improving production. The opening of the stomatal pore is triggered by photosynthetically adequate light, high humidity, and low CO_2_ concentrations, though stomatal closure is assisted by darkness, high temperature, low humidity, and high CO_2_ concentration (Outlaw, 2003; Vavasseur and Raghavendra, 2005; Shimazaki et al., 2007). Moreover, seed morphology, stigmas, and pollen grains revealed a significant micromorphological variation (Ullah et al., 2019). The seeds displayed diversity in shape, dimension, and SEM-based micromorphology. Seed screening provides crucial evidence for investigating the amount of variation observed within the genera and species. The difference in fenugreek seed morphology is exhibited primarily in shape, size, color, *etc*. Seed shape may vary from rhomboid, elliptic, and rectangular to oval (Turki et al., 2013).

## Conclusion

This research indicates that lower and moderate mutagen doses have significantly improved the mean values of morpho-physiological, phytochemical, and quantitative traits of fenugreek. The different treatments of SA induced more mutations as compared with caffeine. Effectiveness and efficiency were highest at the lower mutagenic treatments. Hence, these doses may be employed in future breeding programs to obtain a high frequency of mutations with the least biological damage. The mutagen treatment effectively generated several economically significant mutants with phenotypic alterations. Out of 105 mutants, caffeine and SA respectively produces 51 and 54 individual mutants under seven major phenotypic categories. The broad spectrum of morphological variations/mutations of fenugreek, such as altered plant height, growth habit, leaf morphology, pod, and seed shape, denotes the capacity of mutagens to induce genetic variability. The obtained results confirm that the high potency of the selected mutagenic concentration induced a high phenotypic diversity in the treated population. Thus, high-yielding mutant lines such as tall mutants, bushy mutants, bold seeded pod mutants, *etc*., developed in the present research may be used as mutant varieties directly or as parents in cross-breeding programs for further advancement and stabilization of the traits. Marker-assisted selection of mutants is recommended to check the genetic variability at the DNA level to create beneficial cultivars for farmers.

## Data availability statement

The original contributions presented in the study are included in the article/supplementary material. Further inquiries can be directed to the corresponding author.

## Author contributions

NN: Conceptualization, Methodology, Writing- Original draft preparation, Software; SC: Supervision; NS: Visualization, Data curation, Investigation; NH: Software, formal analysis, Validation, Writing-Reviewing and Editing; NA: Interpretation of data, Revised the manuscript. DA: Data analyzation, Revised and Edited the manuscript. All authors contributed to the article and approved the submitted version.
